# Integrated Biomarker Response of Oxidative Stress Parameters in the Digestive Glands and Gills of Autochthonous and Invasive Freshwater Mussels from the Sava River, Serbia

**DOI:** 10.3390/toxics12100756

**Published:** 2024-10-18

**Authors:** Slavica Borković-Mitić, Bojan Mitić, Jelena S. Vranković, Katarina Jovičić, Slađan Pavlović

**Affiliations:** 1 Department of Physiology, Institute for Biological Research “Siniša Stanković”—National Institute of the Republic of Serbia, University of Belgrade, Bulevar Despota Stefana 142, 11108 Belgrade, Serbia; sladjan@ibiss.bg.ac.rs; 2 Institute of Zoology, Faculty of Biology, University of Belgrade, Studentski Trg 16, 11000 Belgrade, Serbia; bojan@bio.bg.ac.rs; 3 Faculty of Technology Zvornik, University of East Sarajevo, Karakaj 34a, 75400 Zvornik, Bosnia and Herzegovina; 4 Department of Hydroecology and Water Protection, Institute for Biological Research “Siniša Stanković”—National Institute of the Republic of Serbia, University of Belgrade, Bulevar Despota Stefana 142, 11108 Belgrade, Serbia; jeca.s@ibiss.bg.ac.rs (J.S.V.); katarina.jovicic@ibiss.bg.ac.rs (K.J.)

**Keywords:** antioxidant parameters, *Unio pictorum*, *Sinanodonta woodiana*, IBR, Sava River, Serbia

## Abstract

In this study, the activity of oxidative stress parameters superoxide dismutase (SOD), catalase (CAT), glutathione peroxidase (GSH-Px), glutathione reductase (GR), and glutathione S-transferase (GST), as well as the concentrations of vitamin E (Vit E) and SH groups in the digestive glands and gills of freshwater mussels *Unio pictorum* and *Sinanodonta woodiana* from the Sava River in Serbia were investigated. These parameters were determined in native and invasive mussels under the same environmental conditions. The activities of GSH-Px and GR and the concentration of Vit E were significantly higher in the digestive glands of the autochthonous species *U. pictorum* than in the invasive species *S. woodiana*, while the CAT activity and the concentration of SH groups were lower. In the gills of *U. pictorum*, GSH-Px activity and Vit E concentration were significantly higher, while CAT, GST, and SH groups were lower. Principal component analysis (PCA) showed that oxidative stress parameters were strictly tissue- and species-specific. In addition, integrated biomarker response (IBR) showed a combined response of enzymatic and non-enzymatic oxidative stress parameters depending on the tissue or species studied, indicating different metabolic activities and behaviors of an autochthonous versus an introduced bivalve species.

## 1. Introduction

In contaminated environments, especially in river ecosystems, organisms are often exposed to a complex mixture of chemical pollutants. Consequently, they must adapt to a certain degree to these unfavorable environmental conditions. Water pollution is a major contributor to oxidative stress in aquatic species, resulting from the redox cycle of pollution. Under the constant influence of pollutants, defense mechanisms are subject to constant variability and adaptability [1]. During evolution, organisms have had to develop strategies at the cellular level to protect themselves from the harmful effects of pollutants such as organic compounds and metals. One of the most important is the antioxidant defense system, whose role is to bind certain proteins and initiate detoxification processes involving metabolism and the elimination of resistant multixenobiotic proteins [2]. The antioxidant defense system protects cells from the damaging effects of oxygen radicals by keeping endogenous reactive oxygen species (ROS) at a relatively low level and reducing the damage caused by their high reactivity. In addition to low-molecular-weight compounds (vitamin E, ascorbic acid, GSH, etc.), the antioxidant protection mechanism of mussels also includes specially adapted enzymes [3]. In general, non-enzymatic antioxidants are more active outside the cell, while enzymatic antioxidants are more active inside the cell [4].

One of these enzymes is superoxide dismutase (SOD), an important enzyme that enables the conversion of superoxide anions (O_2_^•−^) into molecular oxygen (O_2_) and hydrogen peroxide (H_2_O_2_). Its role as an antioxidant is greatly enhanced by its interaction with other enzymes, such as catalase (CAT), glutathione peroxidase (GSH-Px), and glutathione reductase (GR). In addition, the enzyme glutathione S-transferase (GST) is involved in detoxifying xenobiotics [5]. Moreover, non-enzymatic antioxidants, including various vitamins, contribute to the body’s defense mechanisms by neutralizing free radicals, repairing tissue damage, and increasing antioxidant capacity [6]. These components work together to balance oxidants and antioxidants, which is critical to cellular health.

Freshwater mussels are important aquatic inhabitants and serve as sensitive biomarkers for the pollution of aquatic ecosystems. Mussels are stationary, filter-feeding organisms capable of bioaccumulating and concentrating most pollutants, even in relatively low concentrations [7]. Overall, we chose *Unio pictorum* (Linnaeus, 1758), the painter’s mussel, because it is a natural bio-filter and has a large distribution area. It is a medium-sized freshwater mussel from the family Unionidae. The distribution of *U. pictorum* in Europe means that this native species is widespread here. The non-native species *Sinanodonta woodiana* (Lea 1834) is a large species that can reach a length of 12–26 cm and a maximum height of 12 cm. It is an invasive freshwater mussel that is widely distributed worldwide. The lineage of *S. woodiana* that has invaded Europe originates from the Yangtze River basin in China [8].

The various invasive populations of *S. woodiana* have the potential to affect native mussels and other benthic invertebrates by competing for resources such as food, habitat, and hosts and by serving as a source of parasites [9,10,11]. In addition, non-native species such as *S. woodiana* can compete indirectly with native mussels. Studies show that *S. woodiana* can utilize a wider range of host fishes compared to native Anodonta species, including several fish species found in Europe [9,12,13].

Climate change and the introduction of invasive species pose a significant threat to biodiversity [14]. Invasive alien species show remarkable adaptability to the new environments they invade. In addition to adapting to different physico-chemical conditions, these species must also have considerable metabolic plasticity [15]. The ability to cope with environmental challenges, especially those associated with oxidative stress, represents a significant advantage that may increase the adaptability and invasiveness of certain species. Recent research suggests that many invasive mollusks have a remarkable ability to cope with increased reactive oxygen species (ROS), whether triggered by environmental factors or immune responses [16]. Therefore, further research into the resilience of emerging invasive species to experimental oxidative stress could provide valuable insights into their invasion potential and lead (Pb) to effective management strategies [16].

The main objective of this work is to compare the physiological response of native and invasive bivalves of two Unionidae species concerning their ability to withstand the demands of the environment they inhabit at a given level of pollution. To analyze that physiological response, the following oxidative stress parameters were examined: the activities of superoxide dismutase (SOD, EC 1.15.1.1), catalase (CAT, EC 1.11.1.6), glutathione peroxidase (GSH-Px, EC 1.11.1. 9), glutathione reductase (GR, EC 1.6.4.2), and glutathione S-transferase (GST, EC 2.5.1.18), as well as the concentrations of vitamin E and the SH groups in the native autochthonous freshwater mussel *U. pictorum* and the invasive non-native species *S. woodiana*.

## 2. Materials and Methods

### 2.1. Description of the Site and Sampling

The freshwater mussels *U. pictorum* (n = 10) and *S. woodiana* (n = 10) were collected in August from the Sava River in Šabac (44°46′17.2″ N and 19°42′16.1″ E). This site was selected due to its urban environment, which is subject to significant anthropogenic influences. The Sava River is influenced by both municipal and agricultural wastewater [17]. The mussels were collected by diving, while the water samples were taken from a depth of 0.5 m. On-site analyses included measurements of water temperature, pH, and dissolved oxygen using portable instruments. Alkalinity was determined by titration with an automatic burette immediately after sampling in a field laboratory on the boat. Only sexually mature mussels of similar size were selected for the study, and all samples were stored on ice after collection.

Water samples are routinely collected by the Hydrometeorological Service of the Republic of Serbia, which determines various physical and chemical parameters relevant to this study. The water quality was assessed following Serbian regulations established by the International Commission for the Protection of the Danube River [18]. The environmental quality standards established following the EU directives [19] must not be exceeded to meet the criteria for good chemical status.

### 2.2. Processing of the Tissue and Biochemical Analyses

Each individual’s digestive glands and gills (n = 10 for each species) were dissected on ice, dried, weighed, and frozen in liquid nitrogen (−196 °C) immediately after collection on board and stored at −80 °C until analysis. Tissue preparation and biochemical analyses (protein concentration, activities of SOD, CAT, GSH-Px, GR, GST, and the concentration of vitamin E and SH groups) were determined according to a previous work [20].

Protein electrophoretic profiles were examined by the standard method of sodium dodecyl sulphate polyacrylamide gel electrophoresis (SDS-PAGE) [21]. The principle of this method is based on an anionic detergent SDS, which denatures the proteins and separates them on the gel according to their molecular weight. After casting and polymerizing the gels, the sample, previously mixed with a sample buffer containing SDS and mercaptoethanol, was applied to the resulting sample spaces. The sample volume used depended on the protein concentration. For the electrophoretic analysis of the proteins, we used samples from both tissues (digestive glands and gills) of the freshwater mussels *U. pictorum* (Up) and *S. woodiana* (Sw). This way, differences between the species and a possible polymorphism within the species could be determined. After separation, the gels were stained with Coomassie Brilliant Blue R250 and decolorized in the corresponding counters. The protein profiles obtained represent a qualitative result, indicating differences between the species studied.

The electrophoretic profiles of SOD were analyzed using the nitroblue tetrazolium (NBT) method [22]. This technique of native electrophoresis in combination with photochemical NBT detection allows the simultaneous characterization of different SOD isoforms. First, gels were immersed in an NBT solution for 30 min and then incubated in a suitable buffer with shaking. The volume of the applied sample was adjusted according to the protein concentration. The gel was then exposed to light until it developed a blue hue, with colorless zones indicating the presence of SOD bands. The electrophoretic profiles of SOD were obtained from two tissues—the digestive glands and the gills—of the freshwater mussels *U. pictorum* (Up) and *S. woodiana* (Sw). These profiles provide qualitative insight into the differences between the enzyme properties of these two species.

### 2.3. Statistical Analyses

The values of the measured parameters are given as mean ± standard deviation (SD). All data were tested for normality using the Kolmogorov–Smirnov test (n < 50) and for homoscedasticity using Levene’s test. Differences in oxidative stress parameters between tissues were tested with an independent-sample *t*-test, and *p* < 0.05 was accepted as significant. Statistical analyses were performed using STATISTICA (v. 12.5, Paolo Alto, CA, USA) and SPSS (v. 25, Armonk, NY, USA). Star plots were drawn with Origin Lab (v. 2021, Northampton, MA, USA).

Principal component analysis (PCA) was employed to determine the key variables influencing the variations in the oxidative stress parameters examined [23]. The dataset consisting of these parameters underwent unrotated PCA, utilizing an eigenvalue threshold of greater than >1 for factor extraction, while variables with factor loadings exceeding >0.5 were considered for factor interpretation. This analysis aimed to identify the variables that most significantly distinguished between the digestive glands and gills of *U. pictorum* and *S. woodiana*, as well as the differences between the two tissues in both species. Additionally, PCA was conducted to differentiate the studied tissues and species at the factor level.

The unrotated data matrix was then subjected to varimax rotation after the first step to reduce the number of means and clarify possible relationships between variables. Subsequently, the extraction sums of the squared loadings, the rotation sums of the squared loadings, and the contribution of the studied parameters in each tissue of the two studied species were determined. The Kaiser–Meyer–Olkin index and Bartlett’s test were used to confirm the adequacy of the sample and model, respectively. For all tests used, *p* < 0.05 was considered statistically significant.

Antioxidant biomarkers were combined into a stress index called Integrated Biomarker Response (IBR), described by Beliaeff and Burgeot [24] and modified by Devin et al. [25]. This method provides both a graphical synthesis of the different biomarker responses and a numerical value that integrates all these responses at once. The IBR is the sum of the areas defined by the n biomarkers arranged in a radar plot. This index was calculated for both tissues of the two species as follows: The individual areas Ai connecting the i-th and the (i + 1)-th radius coordinates of the radar plot were calculated according to the following formula:

Ai = 1/2 sin (2π/n) Si Si + 1

where Si and Si + 1 represent the individual biomarker scores (calculated from standardized data) and their successive star plot radius coordinates, and n indicates the number of radii the biomarkers used in the survey. The antioxidant biomarkers used for the IBR index calculation were ranked clockwise according to their hierarchy in ROS detoxification: SOD, CAT, GSH-Px, GR, GST, Vit E, and SH groups. The IBR index was then calculated according to the following formula:

$${\mathrm{IBR}}_{\mathrm{tissue}/\mathrm{species}}=\sum_{i=1}^{n}Ai$$

## 3. Results

The physico-chemical parameters are listed in Table S1 of the Supplementary Material and were measured during sampling. Water and air temperature, dissolved oxygen, alkalinity, carbonates, and bicarbonates, total alkalinity, pH, conductivity, ammonium (NH_4_), nitrites (NO_2_), nitrates (NO_3_), organic and total nitrogen (N), orthophosphates (PO_4_), total phosphorus (P), silicates (SiO_2_), calcium (Ca), magnesium (Mg), chlorides (Cl), sulphates (SO_4_), dissolved iron (Fe), manganese (Mn), zinc (Zn), copper (Cu), total chromium (Cr), Pb, cadmium (Cd), nickel (Ni), arsenic (As), organochlorine pesticides, triazine-based herbicides, and PCBs were measured. Supplementary Table S1 shows that the concentration of the measured parameters in the water of the Sava River was within the normal limits according to Serbian and European standards.

The activities of antioxidant enzymes in the digestive glands and gills of the freshwater bivalves *U. pictorum* and *S. woodiana* are shown in Table 1. The activities of GSH-Px and GR and the Vit E concentration were significantly higher in the digestive glands of *U. pictorum* than in *S. woodiana* (*p* < 0.05), while CAT activity and the concentration of SH groups showed an opposite trend. In the gills of *U. pictorum*, GSH-Px activity and Vit E concentration were significantly higher, while CAT, GST, and SH groups were lower. The concentration of vitamin E was significantly higher in the digestive glands than in the gills, and the same was true for the concentration of the SH groups (Table 1).

**Figure 1:**
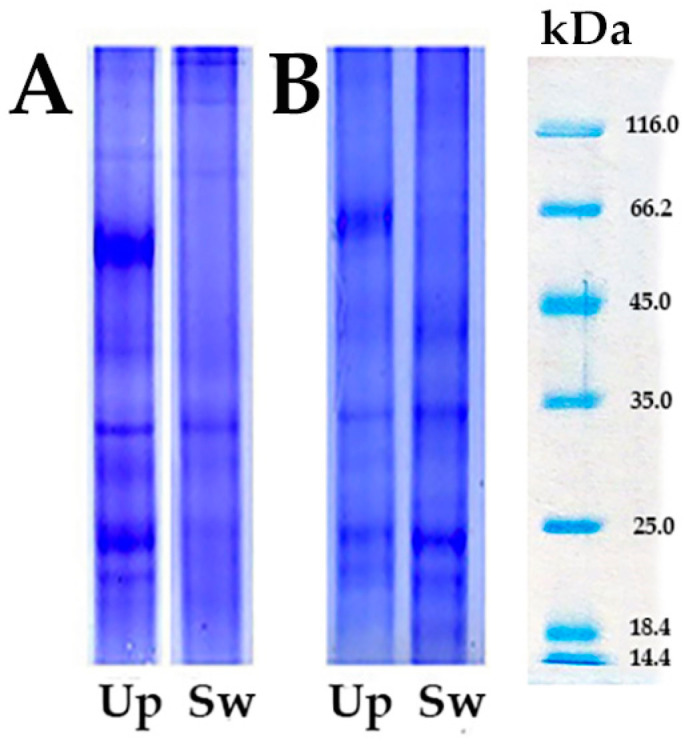
The 1D-SDS-PAGE analysis of proteins with Coomassie blue stained in the (**A**) digestive glands and (**B**) gills of the freshwater mussels *U. pictorum* (Up) and *S. woodiana* (Sw).

One-dimensional 1D-SDS-PAGE of proteins in the digestive glands (Figure 1A) and gills (Figure 1B) of the mussels *U. pictorum* and *S. woodiana* was performed to compare the protein profile of the studied mussels in two different tissues. The obtained electrophorograms visually confirm significant differences in the protein profile of the two species and between the tissues of the studied freshwater mussels.

**Figure 2:**
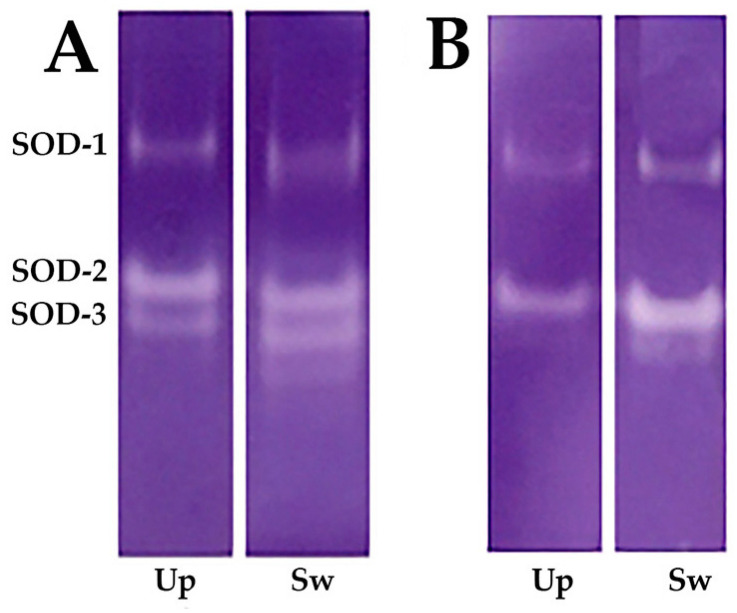
Superoxide dismutase (SOD) electrophoresis in the (**A**) digestive glands and (**B**) gills of the freshwater mussels *U. pictorum* (Up) and *S. woodiana* (Sw).

SOD activity was analyzed directly on the gels after native electrophoresis, using the NBT method (Figure 2). Electrophoretic analysis of SOD from *U. pictorum* and *S. woodiana* in the two tissues examined, the digestive glands (Figure 2A) and the gills (Figure 2B), showed differences in the number of bands, indicating that SOD is significantly different in the digestive glands and the gills. In the digestive glands, three bands were detected according to their molecular weight (Mw): the first band SOD-1, the second band SOD-2, and the third band, which is a unique isoform of SOD-3 that only occurs under polluted environmental conditions (Figure 1A). SOD electrophoresis in the gills (Figure 2B) was different; only two bands (SOD-1 and SOD-2) were observed in both species. The detected isoenzymes of SOD (SOD-1, SOD-2, and SOD-3) are soluble SOD enzymes that can be easily resolved by native gel electrophoresis. These include the cytosolic Cu- and Zn-containing SOD-1, the mitochondrial manganese-containing SOD-2, and the cytosolic manganese-containing SOD-3.

**Figure 3:**
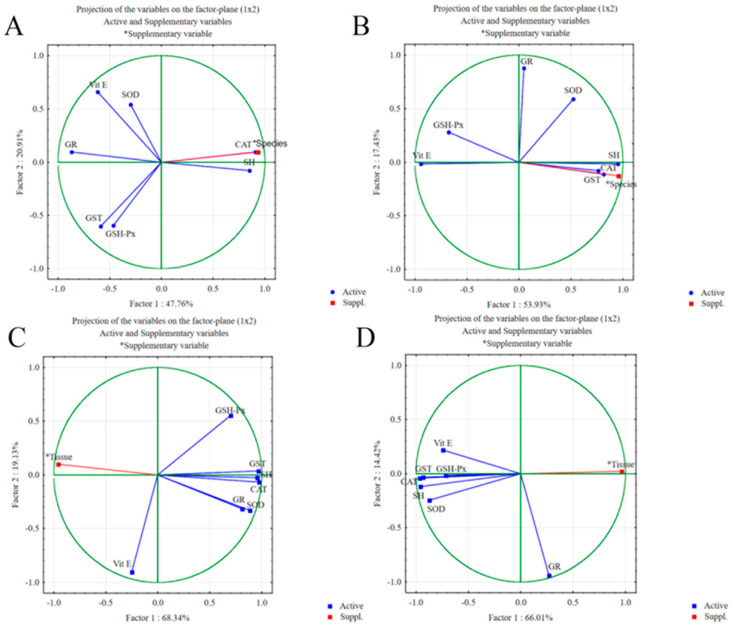
Contribution of oxidative stress parameters based on correlations in the differences between (**A**) the digestive glands of *U. pictorum* and *S. woodiana*; (**B**) the gills of *U. pictorum* and *S. woodiana*; (**C**) the digestive glands and gills of U. pictorum; and (**D**) the digestive glands and gills of *S. woodiana*. Data are given as mean ± SD. *t*-tests for independent samples were performed to seek differences between groups. A minimum significance level of *p* < 0.05 was accepted (significantly different values are marked with *, indicating differences between the digestive glands or gills of *U. pictorum* and *S. woodiana*).

The first step of the PCA analysis was to determine which oxidative stress parameters contributed most to the differences between the tissues and species studied. Figure 3 shows the graphical representation of the PCA results for the digestive glands and gills of the two species studied (A and B) and for the digestive glands and gills of *U. pictorum* and *S. woodiana* (C and D).

The most influential oxidative stress parameters contributing to the differences in the digestive glands of *U. pictorum* and *S. woodiana* are the CAT, GR, and SH groups, with a factor of 1, and Vit E, GST, and GSH-Px, with a factor of 2 (Table 2).

The total variance explained by these two factors is 68.67%. Factor 1 explains 47.76%, and factor 2 explains 20.91% of the total variance. It is also evident that species as an additional variable contributes strongly to the differences, which is consistent with the known differences between species. Similar results were obtained when comparing PCA results of gills between two species (Figure 3B). The total variance explained 71.36%, of which factor 1 explained 53.93% and factor 2 explained 17.43%. The parameters that contributed most to the differences in factor 1 are SH groups, Vit E, and CAT activity, and in factor 2, GR, SOD, and GSH-Px activities (Table 2). There is also a large influence of species as an additional variable.

We also performed PCA to test the differences between two tissues within a species. Figure 3C shows the results of PCA between the digestive glands and gills of *U. pictorum*. Factor 1 and factor 2 together explain 87.47% of the total variance. Factor 1 explains 68.34% and factor 2 explains 19.13% of the variance. The parameters that contribute most to the differences between tissues of *U. pictorum* when factor 1 is considered are CAT, GST, and SH groups, and when factor 2 is considered, it is Vit E, GSH-Px, and SOD. There is also a large tissue specificity depending on the additional variable. The PCA results for the digestive glands and gills of *S. woodiana* are shown in Figure 3D. The total variance explained by the two factors is 80.43%. Factor 1 explains 66.01% of the total variance, and factor 2 explains 14.42% of the total variance. The parameters that contributed most to the differences between these two tissues in *S. woodiana* were SH groups, CAT, and GST for factor 1 and GR, SOD, and Vit E for factor 2 (Table 2). The results of PCA analysis confirm that oxidative stress parameters are strictly tissue- and species-specific.

**Figure 4:**
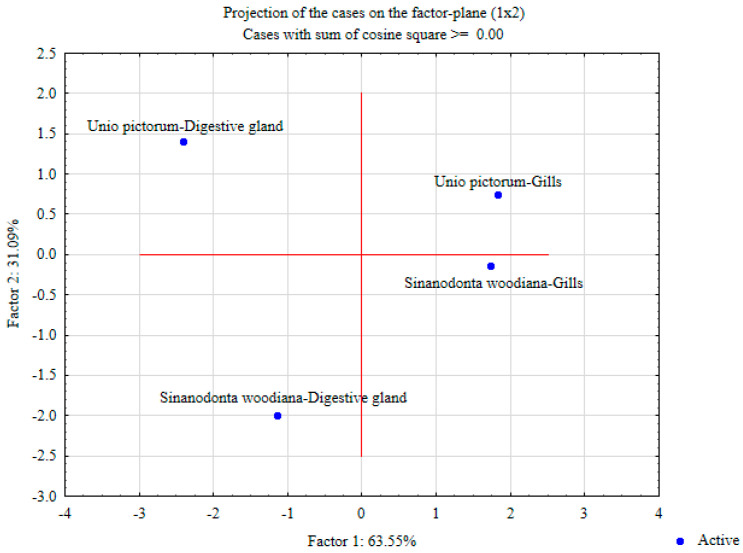
Projection of cases to the factor level between the species and tissues studied.

Figure 4 shows the results of the PCA with the included analysis of all investigated parameters in the digestive glands and gills of the two species; 94.64% of the total variance is explained. Of this, factor 1 explains 63.55% and factor 2 explains 31.09% of the total variance. The results shown in the coordinate system clearly show that there is a strong separation between the analyzed species for factor 1, e.g., digestive glands and gills of *U. pictorum* lie above the x-axis and gills of *S. woodiana* below the x-axis. At the same time, there is a strong separation between the examined tissues concerning factor 2, e.g., the digestive glands of both species lie on the left side of the y-axis and the gills on the right side.

After this step, we performed a Varimax rotation in the PC analysis for each tissue and species separately. The total extractions and rotations as well as the sums of the squared loadings for both tissues and both species are shown in Table 3.

**Figure 5:**
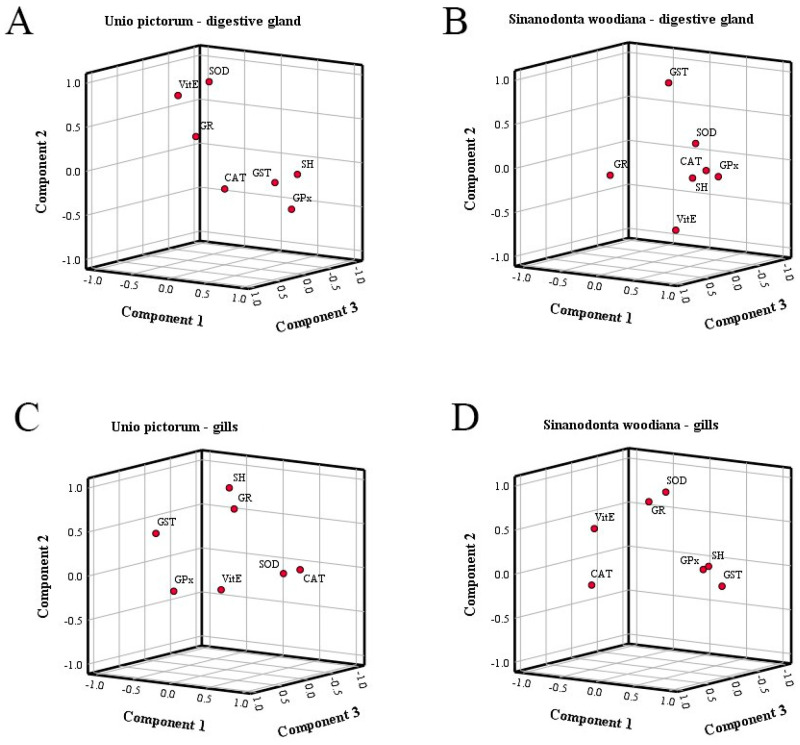
Component plots in rotated space in the digestive glands of (**A**) *U. pictorum* and (**B**) *S. woodiana* and in the gills of (**C**) *U. pictorum* and (**D**) *S. woodiana*.

Table 3 shows a rotated component matrix in the digestive glands and gills of *U. pictorum* and *S. woodiana* using PCA as the extraction method and Varimax with Kaiser normalization as the rotation method. This analysis aimed to determine the variables that contributed the most after rotation in each group to reduce the number of descriptors and clarify possible relationships between variables. The analysis was performed in all cases using three components (factors). In the digestive glands of *U. pictorum*, component 1 extracted GST, GSH-Px, and CAT, component 2 extracted SOD, Vit E, and GR, and component 3 extracted GR and SH groups and CAT as dominant parameters for oxidative stress. In the digestive glands of *S. woodiana*, component 1 extracted SH, CAT, and GSH-Px, component 2 extracted GST, Vit E, and SOD, and component 3 extracted GR, Vit E, and SH groups. Similarly, PCA in the gills of *U. pictorum* extracted the variables as follows: component 1 extracted SOD, CAT, and GST, component 2 extracted SH, GR, and GST, and component 3 extracted GR, SH, and CAT. In the gills of *S. woodiana*, component 1 extracted SH groups, GST, and Vit E, component 2 extracted SOD, GR, and Vit E, and component 3 extracted CAT, SOD, and Vit E. The 3D graphical distribution of all oxidative stress parameters in each tissue of both species is shown in Figure 5 as a component plot in rotated space.

It is obvious that PCA analysis in the digestive glands of *U. pictorum* mainly distinguishes the enzymatic components SOD, GST, and GSH-Px and the non-enzymatic component Vit E. In the digestive glands of *S. woodiana*, these are GR, GST, and Vit E. In the gills of *U. pictorum*, the PCA separated GSH-Px, SOD, and CAT as well as non-enzymatic SH. Finally, in the gills of *S. woodiana*, these were CAT, GST, and SOD and non-enzymatic SH. In both tissues of *U. pictorum* and *S. woodiana*, GST, SOD, and GSH-Px are emphasized as the most common enzymes, but when considering the digestive glands of both species, the dominant non-enzymatic component is Vit E in the gills the SH groups. The PCA results show that there is a difference in oxidative stress parameters that contributes to the differences between the tissues of the two species studied compared to the tissues of each species.

**Figure 6:**
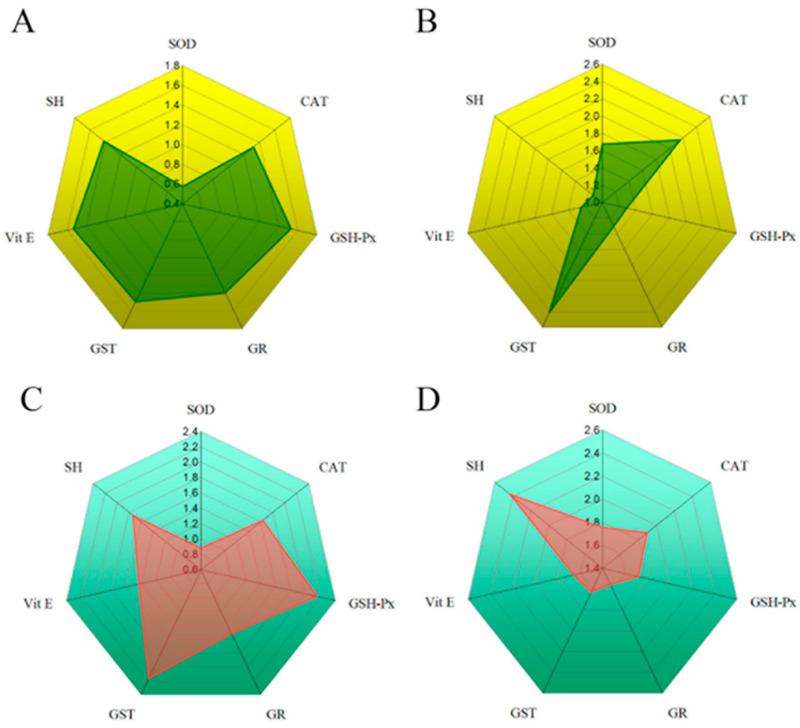
Radar plots of the integrated biomarker response (IBR) of individual oxidative stress parameters in the digestive glands of (**A**) *U. pictorum* and (**B**) *S. woodiana* and in the gills of (**C**) *U. pictorum* and (**D**) *S. woodiana*.

The radar plots of IBR for the digestive glands and the gills of *U. pictorum* and *S. woiodiana* are shown in Figure 6. The calculation of IBR in the digestive glands of *U. pictorum* shows the impact of oxidative stress parameters in the following order: Vit E > GSH-Px > GST >> SH > GR > CAT > SOD. As we can see, Vit E has the highest (1.421) and SOD (0.575) the lowest influence on the IBR value. In the digestive glands of *S. woodiana*, GST has the highest IBR value (2.414), followed by CAT, SOD, GSH-Px, Vit E, and SH. In the gills of *U. pictorum*, the highest IBR value was calculated for GST (2.175), followed by GSH-Px, SH, CAT, GR, Vit E, and SOD. In the gills of *S. woodiana*, the IBR values are as follows: SH (2.436), then CAT > SOD > GSH-Px > Vit E > GST > GR.

**Figure 7:**
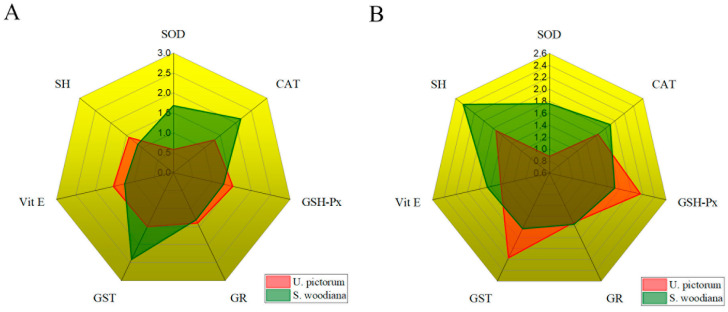
Radar plots of the overlap of the integrated biomarker response (IBR) between *U. pictorum* and *S. woodiana* in (**A**) the digestive glands and (**B**) the gills.

As we can see, the dominant IBR response in the digestive glands of *U. pictorum* is Vit E, while in the digestive glands of *S. woodiana* the highest IBR is GST. In the gills of *U. pictorum*, the highest IBR was calculated for GST, and in *S. woodiana* it was for SH. The IBR results show a combined response of enzymatic and non-enzymatic oxidative stress parameters, depending on the tissue or species studied. Figure 7 shows the overlapping areas with the calculated IBR for both species in both tissues.

## 4. Discussion

Sessile species have limited opportunities to escape stressful conditions and must either withstand the challenges of the environment or become extinct. Tolerating difficult conditions requires either a physiological response or a whole organism response. For this reason, freshwater and marine mussels are often used as bioindicator organisms [26]. In mussels, antioxidant activity is influenced by many factors: (a) anaerobic conditions lead to a decrease in enzyme activity and lipid peroxidation, which returns to normal when oxygen is supplied [27]; (b) reproduction influences the increase in antioxidant activity in the season from March to April, followed by a gradual decrease in spring when food supply and temperature increase [28]; (c) with age, sensitivity to oxidative effects increases as oxidative capacity is weakened, leading to an increase in lipid peroxidation [29].

The hemolymph of mussels contains several protective antioxidant enzymes—SOD, CAT, and GSH-Px—which protect against free radicals. Metals and organic xenobiotics taken up by hemocytes concentrate in their endolysosomal system, leading to either detoxification or excessive formation of ROS [30].

The change in SOD activity is a good biomarker for environmental pollution, as it responds relatively quickly to environmental stressors. In polluted areas, the activity of SOD increases significantly in the gills of the mussel *Mytilus galloprovincialis* but not in the digestive glands [31]. This contradicts our results but indicates that the tissues show a specific reaction to certain pollutants, which are absorbed to a greater or lesser extent in the various tissues. Our data are consistent with the earlier results of Cossu et al. [32] for *Unio tumidus*. These authors showed that the gills are more sensitive to oxidative stress, leading to an inhibition of antioxidant parameters, and that enzyme activity is lower in the gills than in the digestive glands. The great sensitivity of the gills is that they are directly exposed to oxygen during respiration and filtration of food, resulting in greater exposure to various pollutants and higher dissolved oxygen pressure. Differences in tissue expression have been found between the digestive glands and the gills, with the digestive glands generally having a higher metabolic rate. The digestive glands are of particular importance as they are involved in most biotransformation processes and the redox cycle and show a wide variation in activity levels, which complicates the interpretation of the results in contrast to those of the gills [20]. The antioxidant system of the digestive glands is also influenced by internal factors (e.g., diet, spawning time) [33]. In our experiments, CAT activity was found to be higher in the digestive glands than in the gills of the two mussels studied. Comparing the CAT activity in the mussels examined in our study, the activity in *S. woodiana* was significantly higher. Gills are exposed to high O_2_ concentrations due to their respiratory function and therefore require an efficient enzymatic mechanism against the large amount of free radicals produced when filtering large amounts of water to ensure sufficient oxygen [20]. An increase in CAT activity above normal levels is an indicator of an increase in H_2_O_2_ levels in the aquatic environment, very often due to anthropogenic pollution. The physiological, ecological, and biological characteristics of the species are the reasons for its success and give it advantages over the native unionid species. *Sinanodonta woodiana* is in an accelerated process of dispersal and appears to be less demanding and better adapted to water pollution than native species [34].

Benthic mollusks can serve as good indicators of biological stress caused by heavy metals and hydrocarbons, as the activity of SOD and CAT increases in their presence [35]. In the work of Angel et al. [36], CAT activity in the mussel *Dontax trunculus* was determined as an indicator of biological stress. They concluded that CAT activity is always higher in polluted water than in unpolluted or less polluted habitats. In the presence of xenobiotics such as heavy metals, polycyclic aromatic hydrocarbons (PAHs), and polychlorinated biphenyls (PCBs), there is an accumulation of endogenous superoxide radicals [37]. To prevent the increase in superoxide radicals, the cell increases the activity of SOD and CAT.

Zhang et al. [38] observed the response of biomarkers SOD, CAT, GSH-Px, and GSH in the mussel *Clamys farreri* to the presence of the metals Cu, mercury (Hg), and Pb in the water. Exposure to Pb leads to a significant inhibition of GSH-Px activity. Cu did not affect the antioxidant defense system, except GSH-Px, which was inhibited by 8.83%. Of all the enzymes examined in this study, only GSH-Px was significantly inhibited by Hg (33.2%). The inhibition of GSH-Px by Hg is probably due to its high affinity for thiols. Inorganic Hg binds to thiol-containing proteins of different molecular weights (glutathione, cysteine, and albumin). Hg competes with GSH for thiol groups and forms GS-Hg-SG, causing a strong reduction of GSH in the fish liver. Intracellular thiol depletion due to Hg binding disrupts the composition and activity of proteins in the cell and can contribute to oxidative stress [39]. Metals such as Cd, Fe, Cu, and Pb cause a decrease in GSH-Px activity and GSH levels at LC50 concentrations, and lipid peroxidation occurs in *Perna perna* [40].

Comparing the GSH-Px activity in the mussels examined in our study, the activity was significantly higher in *U. pictorum*, both in the digestive glands and the gills. The activity of CAT and GSH-Px plays an important role in cellular homeostasis by preventing the formation of radical intermediates in the reduction of H_2_O_2_ and organoperoxides. GSH-Px activity in mussels can be induced by environmental pollutants [41]. The positive correlation between SOD and GSH-Px in the work of Férnandez et al. [42] suggests that GSH-Px, which is important for H_2_O_2_ degradation pathways, counteracts oxidative attack by peroxide in a coordinated action with SOD. Other studies that show a similar relationship include Borković et al. [43] in *M. galloprovincialis* from the Adriatic Sea, where high SOD activity is associated with increased GSH-Px and GR activities.

GR has rarely been used in biomonitoring, although it is very important for the maintenance of GSH/GSSG homeostasis under conditions of oxidative stress. In our study, GR activity was statistically significantly higher in the digestive glands of *U. pictorum* compared to *S. woodiana*, while there was no significant difference in the gills. In the work of Doyotte et al. [44], antioxidant defense enzymes, GSH, and lipid peroxidation were observed in the digestive glands and gills of the freshwater mussel *U. tumidus* after three days of exposure to Cu (30 μgL^−^^1^) and thiram (100 μgL^−^^1^). Changes were observed in the activity of GR, Se-dependent GSH-Px, and GSH in the digestive glands and gills, while the activity of SOD, CAT, and total GSH-Px did not change. The decrease in some antioxidant defense parameters suggests that the mussels are exposed to oxidative stress as a result of various environmental factors. The activity of GR changed during the seven-day chemical exposure; this enzyme can be considered a valuable and early indicator of exposure.

GST activity was higher in the digestive glands of *U. pictorum* than in those of *S. woodiana*, while the situation was reversed in the gills and GST activity was higher in *S. woodiana* (Table 1). High GST activity in the digestive glands is associated with detoxification. The digestive glands are very important tissues for the absorption of xenobiotics and are involved in several biotransformation processes [45]. A significant induction of GST activity was found by Lu et al. [46] under the influence of complex pollutants (PAHs, PCBs, organochlorine pesticides, and heavy metals) in *Carassius auratus* in Lake Taihu. The GST enzyme is useful in conjugation reactions in mussels exposed to organic contaminants. A significant correlation was found between GST and the concentration of heavy metals (Hg, Cd, and Pb), which has also been found in numerous other studies [33]. Since metals are not a natural substrate for this enzyme, it is assumed that the increase in GST activity in the gills is a response to the oxidative stress caused by metals. The high GST activity in the gills could compensate for the low activity of CAT, which was confirmed by Lima et al. [47]. In the work of Liu et al. [48], a significant positive correlation between GST and GSH-Px was found, which can be explained by the coordinated expression of total GST and its peroxidase isoform. Similar correlations between antioxidant enzymes were observed in the work of Borković et al. [43].

The comparison of vitamin E concentrations in the mussels we examined showed that *U. pictorum* had significantly higher vitamin E concentrations in both the digestive glands and the gills (see Table 1). Barim and Karatepe [49] investigated how environmental pollution affects the concentrations of antioxidant vitamins and malondialdehyde (MDA) in the tissues of freshwater crayfish (*Astacus leptodactylus*). The results indicate differences in metabolic activity depending on the environmental conditions and the sex of the organisms studied. In polluted areas, the crayfish showed increased MDA levels, while the concentrations of vitamins E, A, C, and β-carotene decreased. This trend is probably due to the increased concentrations of heavy metals in these areas. 

The concentration of SH groups was significantly higher in both the digestive glands and the gills of *S. woodiana* than in the native *U. pictorum*. Protein SH groups bind metals, and metal ions’ fate depends on the thiol-containing molecule content. Cd has a high affinity for SH groups and binds low-molecular antioxidant thiol-containing peptides such as glutathione and metallothionein. The depletion of the pool of antioxidant peptides due to their association with Cd is the most important mechanism for causing metal-induced oxidative stress [50].

Electrophoresis under native conditions with photochemical NBT disclosure allowed the characterization of the SOD isoforms. The observed parameters are well suited for biomonitoring, especially the SOD-3 isoform identified in the digestive glands of both mussel species (see Figure 1). According to Manduzio et al. [51], two primary bands were identified, as SOD-1 and SOD-2, while SOD-3 was consistently present and highly expressed in the digestive glands of mussels collected from polluted sites. The third band in the electrophoretic profile, designated SOD-3, was detected in the digestive glands of both *U. pictorum* and *S. woodiana*. Manduzio et al. [51] suggest that the SOD-3 isoform may be related to the degree of pollution. Our results indicate that variations in SOD expression patterns in *U. pictorum* and *S. woodiana* could serve as an effective tool for environmental monitoring.

In studies with multiple biomarkers, IBR has proven to be a practical tool to better understand complex outcomes. In this way, it is more informative than analyzing the response of individual biomarkers [25]. Using the IBR index, parameter comparisons were made between the two mussels studied and two metabolically different tissues based on two visual criteria: size and geometric shape of the polygonal area. Figure 7 shows the areas of overlap with the calculated IBR for both species in both tissues. The predominant IBR response in the digestive glands of *U. pictorum* is Vit E, while in the digestive glands of *S. woodiana,* the highest IBR shows GST. In the gills of *U. pictorum,* the highest IBR was calculated for GST, and in *S. woodiana* it was for SH groups. The IBR results show a combined response of enzymatic and non-enzymatic parameters of oxidative stress, depending on the tissue or species studied. From the graphical representation (Figure 6), it can be concluded that in the digestive glands and gills of *U. pictorum* (Figure 6A,C), there is a more even redistribution of IBR to a larger number of biomarkers, while in *S. woodiana* there are biomarkers that are subjected to greater pressure in the biomarker response in both tissues (Figure 6B,D). The predominant biomarker in the response to environmental stress appears to be the enzyme GST in the gills of *U. pictorum* and the digestive glands of *S. woodiana*. In both species and both tissues, CAT and GSH-Px appear as significant biomarkers, indicating an increased presence of H_2_O_2_. The response of the low-molecular-weight components of antioxidant protection Vit E and SH groups is also significant in the digestive glands and gills of both species studied. Star plots were used in this study as one of the possible tools to visualize biological effects. Thus, star plots can be used as a useful graphical tool for exploratory data analysis in a multi-biomarker approach.

## 5. Conclusions

The present study shows that the digestive glands and gills of the autochthonous *U. pictorum* and the invasive mussel *S. woodiana* respond differently to environmental influences, including pollution. One of the possible reasons for the greater adaptability of this invasive species could be an increased ability to cope with oxidative stress responses, which makes it more adaptable to new environments. Exposure to pollutants and other environmental influences leads to potential problems for both native and non-native mussel species. *U. pictorum* responded with an increase in GSH-dependent enzymes in the digestive gland, while *S. woodiana* showed a strong response, with a marked increase in CAT activity in the digestive glands and gills. *S. woodiana* may have greater tolerance to the same levels of pollution and greater adaptability compared to the native species *U. pictorum*. PCA analysis showed that oxidative stress parameters are strictly tissue- and species-specific, while IBR analysis confirmed different defense mechanisms between these two species. It is expected that analyzing the plasticity of antioxidant responses in native and invasive mussels under the same environmental stresses may contribute to the understanding of the underlying mechanisms.

## Figures and Tables

**Table 1:** Activities of superoxide dismutase (SOD, U/mg protein), catalase (CAT, U/mg protein), glutathione peroxidase (GSH-Px, U/mg protein), glutathione reductase (GR, U/mg protein), and glutathione S-transferase (GST, U/mg protein), as well as the concentrations of vitamin E (Vit E, μg/g wet mass) and sulfhydryl groups (SH, μmol/g wet mass) were determined in the digestive glands and gills of freshwater mussels *U. pictorum* and *S. woodiana* from the sampling site Šabac, Sava River, Serbia.

	*Unio pictorum*	*Sinadonta woodiana*	*Unio pictorum*	*Sinadonta woodiana*
	Digestive Glands		Gills	
SOD	20.14 ± 2.58	20.25 ± 1.92	14.02 ± 1.72	15.55 ± 1.52
CAT	47.91 ± 9.17	182.04 ± 32.77 *	14.79 ± 1.61	24.64 ± 5.39 *
GSH-Px	5.65 ± 0.96	4.63 ± 1.18 *	4.61 ± 0.73	3.26 ± 0.87 *
GR	7.09 ± 1.92	3.52 ± 0.98 *	4.07 ± 1.23	3.92 ± 0.72
GST	1188.53 ± 283.56	1006.64 ± 169.89	147.93 ± 21.59	212.16 ± 45.34 *
Vit E	45.19 ± 3.67	41.59 ± 1.93 *	45.80 ± 1.68	38.70 ± 0.80 *
SH	193.51 ± 21.49	247.54 ± 26.17 *	51.48 ± 15.32	124.93 ± 18.12 *

Data are given as mean ± SD. *t*-tests for independent samples were performed to seek differences between groups. A minimum significance level of *p* < 0.05 was accepted (significantly different values are marked with *, indicating differences between the digestive glands or gills of *U. pictorum* and *S. woodiana*).

**Table 2:** Contributions of the variables based on correlations. The variables with the largest contribution are in bold and marked with an asterisk. UP—*U. pictorum*; SW—*S. woodiana*. * Significantly different correlations.

	Variable Contributions, Based on Correlations							
	Digestive Glands UP vs. SW		Gills UP vs. SW		Digestive Glands vs. Gills UP		Digestive Glands vs. Gills SW	
Variable	Factor 1	Factor 2	Factor 1	Factor 2	Factor 1	Factor 2	Factor 1	Factor 2
SOD	0.026732	0.198614	0.071617	0.284501 *	0.163943	0.082867 *	0.165069	0.060479 *
CAT	0.246672 *	0.006353	0.175202 *	0.010229	0.198941 *	0.003619	0.201302 *	0.001700
GSH-Px	0.064500	0.241708 *	0.121943	0.065131 *	0.102136	0.226355	0.111350	0.000279
GR	0.226706 *	0.006053	0.000551	0.634269 *	0.137021	0.074238	0.015824	0.874596 *
GST	0.103563	0.247665 *	0.153383	0.005408	0.194789 *	0.001021	0.187850 *	0.001214
Vit E	0.114340	0.295195 *	0.237016 *	0.000196	0.012913	0.611293	0.119525	0.047247 *
SH	0.217487 *	0.004411	0.240289 *	0.000266	0.190257 *	0.000608	0.199079 *	0.014484

**Table 3:** Rotated component matrix for the digestive glands and the gills of *Unio pictorum* and *Sinanodonta woodiana*. Extraction method: principal component analysis. Rotation method: Varimax with Kaiser normalization. Variables contributed the most in each group.

	*Unio pictorum*—Dig. Glands				*Sinanodonta woodiana*—Dig. Glands		
	Component				Component		
	1	2	3		1	2	3
GST	0.823			SH	0.807		0.380
GSH-Px	0.816			CAT	0.742		
SOD		0.978		GPx	0.721		
VitE		0.785		SOD		0.318	
GR		0.477	0.787	GST		0.896	
SH			0.773	ViE		0.813	0.439
CAT	0.526		0.742	GR			0.920
	***Unio pictorum*** **—Gills**				***Sinanodonta woodiana*** **—Gills**		
	**1**	**2**	**3**		**1**	**2**	**3**
SOD	0.943			SH	0.880		
CAT	0.857			GST	0.837		
SH		0.935		VitE	0.601	0.451	0.288
GR		0.776		GPx			
GSH-Px			0.950	SOD		0.835	0.316
GST	0.544	0.468	0.595	GR		0.800	
ViE			0.580	CAT			0.958

## Data Availability

All data generated or analyzed during this study are included in this article [and its Supplementary Information file].

## Supplementary Materials

The following supporting information can be downloaded at: https://www.mdpi.com/article/10.3390/toxics12100756/s1, Table S1: Physical and chemical characteristics of the Sava River sampling site.
