# Supplementary material for: Integrated Biomarker Response of Oxidative Stress Parameters in the Digestive Glands and Gills of Autochthonous and Invasive Freshwater Mussels from the Sava River, Serbia
Source: Toxics. 2024 Oct 18;12(10):756. doi: 10.3390/toxics12100756 (PMC11510816; doi:10.3390/toxics12100756)
Supplement: Supplementary file 1 [file toxics-12-00756-s001.zip › toxics-3221037-supplementary.pdf]

Integrated biomarker response of oxidative stress parameters in the digestive gland and gills of autochthonous and invasive freshwater mussels from the Sava River, Serbia

Slavica Borković-Mitić[1]*, Bojan Mitić[2,3], Jelena Vranković[4], Katarina Jovičić[4], Slađan Pavlović[1]

[1] Department of Physiology, University of Belgrade, Institute for Biological Research "Siniša Stanković" – National Institute of the Republic of Serbia, Bulevar Despota Stefana 142, 11108 Belgrade, Serbia; borkos@ibiss.bg.ac.rs

[2] Institute of Zoology, University of Belgrade – Faculty of Biology, Studentski Trg 16, 11000 Belgrade, Serbia; bojan@bio.bg.ac.rs

[3] Faculty of Technology Zvornik, University of East Sarajevo, Karakaj 34a, 75400 Zvornik, Republic of Srpska, Bosnia and Herzegovina; bojan@bio.bg.ac.rs

[4] Department of Hydroecology and Water Protection, Institute for Biological Research "Siniša Stanković" – National Institute of the Republic of Serbia, University of Belgrade, Bulevar Despota Stefana 142, 11108 Belgrade, Serbia; jeca.s@ibiss.bg.ac.rs, katarina.jovicic@ibiss.bg.ac.rs

* Correspondence: borkos@ibiss.bg.ac.rs.; Tel.: (+381112078341)

Supplementary Table S1. Physical and chemical characteristics of the Sava River on the sampling site.

	**River Sava**		**ŠABAC**		
	**Parameter**	**Unit**	Left bank	Middle course	Right bank
**Temperature**	Water temperature	^0C	23,0	22,8	23,0
	Air temperature	^0C	25,6	26,2	26,4
**Oxygen parameters**	Dissolved oxygen	mgO_2/L	6,4	6,5	6,4
**Carbonates, alkalinity and acidity**	Alkalinity	mmol/L	3,7	3,6	3,7
	Total hardness ($CaCO_3$)	mg/L	241	226	186
	CO_2 free	mg/L	3,8	3,3	6,4
	Carbonates (CO_3^{--})	mg/L	0,0	0,0	0,0
	Bicarbonates (HCO_3^-)	mg/L	224	219	224
	Total alkalinity ($CaCO_3$)	mg/L	184	179	183
**pH**	pH	pH-jed.	7,8	7,8	7,7
**Electrical conductivity**	Electrical conductivity	µS/cm	420	421	422
**Dissolved ions**	Total dissolved salts	mg/L		258	
**Nutrients-nitrogen and its compounds**	Ammonium (NH_4-N)	mg/L	0,04	0,06	0,03
	Nitrites (NO_2-N)	mg/L	0,002	0,003	0,002
	Nitrates (NO_3-N)	mg/L	0,50	0,70	0,50
	Organic nitrogen(N)	mg/L	0,8	0,6	0,9
	Total nitrogen (N)	mg/L	1,4	1,3	1,5

**Nutrients-phosphorus and its compounds**	Orthophosphates (PO_4-P)	mg/L	0,054	0,051	0,053
	Total phosphorus (P)	mg/L	0,059	0,063	0,065
**Silicates**	Silicates (SiO_2)-dissolved	mg/L	5	5	6
**Inorganic components-Cations**	Calcium (Ca^{++})	mg/L	69	68	74
**Inorganic components - Anions**	Magnesium (Mg^{++})	mg/L	17	14	17
	Chlorides (Cl^-)	mg/L	8,0	9,0	9,0
	Sulphates (SO_4^{--})	mg/l	22	25	21
**Metals-macro elements**	Iron (Fe)-dissolved	mg/L	0,05	0,05	0,050
	Manganese (Mn)-dissolved	mg/L	<0.01	<0.01	<0.01
**Metals-microelements**	Zinc (Zn)-dissolved	µg/L	<1	<1	<1
	Copper (Cu)-dissolved	µg/L	<1	<1	<1
	Total chromium (Cr) dissolved	µg/L	<1	<1	<1
	Lead (Pb)-dissolved	µg/L	<1	<1	<1
	Cadmium (Cd)-dissolved	µg/L	<0.2	<0.2	<0.2
	Nickel (Ni)-dissolved	µg/L	<1	<1	<1
**Metalloids and nonmetals**	Arsenic (As)-dissolved	µg/L	<1	<1	<1
**Organic compounds**	α–BHC	µg/L	<0.001	<0.001	<0.001
**Organochlorine pesticides**	β–BHC	µg/L	<0.001	<0.001	<0.001
	γ–BHC(Lindan)	µg/L	<0.002	<0.002	<0.002
	Hexachlorobenzene	µg/L	<0.001	<0.001	<0.001
	Heptachlor	µg/L	<0.001	<0.001	<0.001
	Heptachlor-epoxide	µg/L	<0.001	<0.001	<0.001
	Aldrin	µg/L	<0.001	<0.001	0,003
	Endrin	µg/L	<0.002	<0.002	<0.002
	DDE	µg/L	<0.002	<0.002	<0.002
	Dieldrin	µg/L	<0.002	<0.002	<0.002
	p,p'-DDD	µg/L	<0.002	<0.002	<0.02
	p,p'-DDT	µg/L	<0.002	<0.002	<0.002
	o,p'DDT	µg/L	<0.002	<0.002	<0.002
	Methoxychlor	µg/L	<0.003	0,020	<0.003
**Triazine-based herbicides**	Atrazine	µg/L	<0.009	<0.009	<0.009
	Simazine	µg/L	<0.009	<0.009	<0.009
	Propazine	µg/L	<0.009	<0.009	<0.009
**PCBs**	PCB 28	µg/L	<0.001	<0.001	<0.001
	PCB 52	µg/L	<0.001	<0.001	<0.001
	PCB 101	µg/L	<0.001	<0.001	<0.001
	PCB 138	µg/L	<0.001	<0.001	<0.001
	PCB 153	µg/L	<0.001	<0.001	<0.001

PCB 180	μg/L	<0.001	<0.001	<0.001
PCB 194	μg/l	<0.001	<0.001	<0.001

* Environmental Quality Standards according to TNMN standards (ICPDR, 2006)
